# Noncommunicable diseases behavioural risk factors among secondary school adolescents in Urban Cameroon

**DOI:** 10.1186/s12889-024-17753-1

**Published:** 2024-02-05

**Authors:** Changoh Marvel Changoh, Lambed Tatah, Desmond Aroke, Dickson Nsagha, Simeon-Pierre Choukem

**Affiliations:** 1 Faculty of Health Sciences, University of Buea, Buea, Cameroon; 2 Health and Human Development (2HD) Research Network, Douala, Cameroon; 3 Medical Research Council Epidemiology Unit, University of Cambridge, Cambridge, CB2 0QQ UK; 4 Faculty of Medicine and Pharmaceutical Sciences, The University of Dschang, Dschang, Cameroon

**Keywords:** NCDs, Behavioural risk factors, Adolescence, Cameroon

## Abstract

Adolescence is a crucial period for noncommunicable disease (NCD) risk factors, and interventions to reduce the NCD burden must target this age group. This study aimed to evaluate the NCD behavioural risk factors in adolescents attending state secondary schools in an urban setting in Cameroon. We conducted a cross-sectional survey using adapted structured questionnaires to assess the prevalence and correlates of NCD behavioural risk factors among adolescents attending selected urban state secondary schools in Douala IV, one of the six subdivisions in Douala, Cameroon. Of the 645 students who completed the study questionnaires, half of them did not have adequate knowledge about NCDs and their risk factors. Only 20% met recommended physical activity levels, nearly half lived sedentary lifestyles, and only 7% ate a healthy diet. Almost half of all participants reported drinking alcohol during the month, while 3% reported cigarette smoking. Participants with inadequate knowledge of NCDs were more likely to have elevated blood pressure values, and males had increased odds of high blood pressure. Contrarily, being male appeared to be protective against overweight and obesity. The odds of being sedentary decreased with age, and the odds of alcohol drinking seemed to grow with a higher maternal level of education. Our survey shows inadequate knowledge about NCDs and a high prevalence of NCD behavioural risk factors in adolescents in urban state secondary schools in Cameroon. These findings predict a higher NCD burden in future adults in the country, reinforcing the need for urgent public health interventions, especially regarding knowledge and sedentary living. Further research is needed to establish the transition of adolescent risk factors to adult disease through life course approaches in these settings.

## Introduction

Noncommunicable diseases (NCDs) such as cardiovascular diseases, type 2 diabetes mellitus, cancers, and chronic respiratory diseases are the leading cause of global morbidity and mortality, disproportionately affecting low- and middle-income countries (LMICs) [1–4]. In 2016, NCDs caused 71% of the 57 million global deaths, with 78% of all NCD-related deaths and 85% of all premature deaths occurring in LMICs [5]. Although LMICs bear the highest NCD burden, NCDs contribute lower proportions of their overall disease burden since infectious diseases and mother and child health challenges dominate [6]. However, LMICs can no longer afford to neglect NCDs because their rapid rate of urbanisation, sedentary living, and consumption of unhealthy diets are making NCDs more prominent in the current epidemiological transition [1, 4]. For example, 43% of deaths in Cameroon are NCD-related [7], and the estimated probability of dying from a major NCD before 70 years is 21.6% [5]. Despite the growing burden, our understanding of NCD risk factors in LMICs remains limited.

The four major NCDs—cardiovascular diseases (CVDs), type 2 diabetes mellitus (DM2), cancers and chronic respiratory disorder—share the following main behavioural risk factors: tobacco use, harmful alcohol consumption, physical inactivity, and unhealthy diet [4, 8–11]. These risk factors are poorly studied in Cameroon, albeit indicative data pointing to their higher prevalence. Almost 9% of youth could be smoking [12]; 10% of adults could be obese; 27% of adults could be physically inactive; and the mean daily salt consumption among adults could exceed 5 g/day [8]. The data on other risk factors and their distributions among different population subgroups are rarely available to inform interventions.

It is dire when the country’s population is mainly young, and its adolescent NCD behavioural risk profile is sparse since NCD-related risky behaviours are known to rise among adolescents and establish patterns that persist into adulthood [13]. For instance, three-quarters of obese adolescents remain obese in adulthood. Up to 95% of adult smokers began smoking before age 18, and adolescents who start consuming alcohol before 15 are five times more likely to abuse alcohol in adulthood and have an increased probability of having an NCD [13].

Given that adolescence is such a crucial period for developing adult NCDs, interventions aimed at reducing the burden of NCDs must include addressing risk factors in this age group. This study aimed to evaluate the NCD behavioural risk factors among adolescents attending state secondary schools in an urban setting in Cameroon. Specifically, we assessed their knowledge of NCDs and risk factors, the prevalence of specific NCD risk factors, and the factors associated with the distribution of NCD-related risk factors in these adolescents.

The research questions were:

1. What is the level of knowledge on NCDs and their risk factors among school adolescents in the Douala IV subdivision?
2. What is the prevalence of NCD risk factors among these adolescents?
3. What is the association of knowledge level and demographic factors with NCD risk factors in these adolescents?

## Methods

### Study design, setting and population

We conducted a cross-sectional survey to assess the prevalence and correlates of NCD behavioural risk factors among adolescents attending urban state secondary schools in Douala, Cameroon. We collected data using structured questionnaires between January and April 2019. We reported the study in accordance with the STROBE guidelines for cross-sectional studies [14].

The study area was a subdivision in Douala (Douala IV), Cameroon, the country’s economic capital, comprising six subdivisions. This study area was chosen as a representative urban administrative unit and because the researchers were familiar with the setting. The selected subdivision had an estimated population of 250,626 inhabitants and a surface area of 55 square kilometres in 2019. There were approximately 44 secondary schools in the area, nine of which were functional state secondary schools. We judged that the state schools would be the most inclusive and selected five from the list of nine, with the probability of selection proportional to size. Selected schools had populations ranging from 95 to 3500 students in the senior classes (fifth, sixth and seventh forms).

The study population was adolescents aged 14 to 19. The inclusion criteria for study participants were: (i) being of the target age and (ii) providing consent and assent to the study. We excluded participants who were (i) not present at the time of the study or (ii) whose questionnaires were incompletely filled.

### Sample size and sampling

We estimated the initial sample size for unknown disease prevalence of 384 participants [15]. Adjusting for design effect by 1.2 and a non-response rate of 10%, the minimum sample size for this study was 507 participants. Students were selected from all five schools with probability proportionate to size, based on the total number of students in form five, lower sixth and upper sixth classes. Classes were randomly sampled within each school, and all students within a selected class at the time of data collection were invited to participate, leading to an initial sample of 700 students.

### Data collection

Data were collected using self-administered questionnaires. These were pre-tested modified Global School-based Health Survey (GSHS) questionnaires [16]. Before administering the questionnaire, consent forms were signed by parents/guardians for all participants and all participants were required to sign a assent forms. Parent/guardian was used to refer to an adolescent’s legal guardian as school adolescents were either living with their parents or legal guardians. The questionnaire was structured in four sections: sociodemographic information; knowledge about NCDs; NCD behavioural risk factors, adapted from the GSHS core module; and a data extraction sheet for entering biological and anthropometric measurements. The questionnaires were pre-tested on 20 students in the target age group who were attending one of the schools not selected for the study. Anthropometric measurements were taken using weight scales and stadiometers, and blood pressure was measured using automatic Omron® sphygmomanometers of appropriate cuff size. All measurements were done following standard operating procedures.

### Variables

We defined three categories of variables: sociodemographic, knowledge, and NCD risk factor variables. The sociodemographic variables were age, sex, parents’ level of education, and household size. Knowledge was defined as inadequate or adequate for overall NCDs and their risk factors. This variable resulted from 42 Yes/No questions entered as ‘1 or 0’, respectively. The total score was 42, and any individual who scored less than the median was considered to have inadequate knowledge.

NCD behavioural risk factors were captured using the Youth Risk Behaviour Survey Analysis (YRBS) [17]. NCD risk factors were harmful alcohol consumption, tobacco use, unhealthy diet, low physical activity, sedentary living and overweight status. We defined harmful alcohol consumption as anyone who had consumed alcohol on any day within the preceding month; low physical activity as anyone who had not been physically active for at least 60 min per day for ≥ 5 days/week; sedentary living as anyone who watched television, played video games or worked on the phone or the computer for ˃2 h a day; a poor diet as not consuming fruits ≥ 5 days/week and vegetables ≥ 5 servings per day; tobacco use as anyone who was a current cigarette smoker or had used tobacco in any other form on at least one day in the preceding month; secondhand smoking as anyone who had been exposed to tobacco smoke on at least one day in the preceding week; overweight and obesity as more than + 1 and + 2 SD respectively, of the BMI for age and sex; elevated blood pressure as systolic pressure ≥ 2 SD for age and sex.

### Statistical analysis

We used R statistical software for data analysis. Descriptive statistics included means with standard deviations for continuous variables and frequency distributions with proportions for categorical variables. We used the chi-squared test to compare proportions and the t-test to compare means. A *p*-value < 0.05 was considered statistically significant. We used logistic regressions to evaluate the relationships between sociodemographic variables and each NCD risk factor.

### Bias

To reduce bias, we used a clustered random sampling approach to select adequate samples while minimising selection bias. We also standardised our data collection procedures and instruments and applied these across all participants. For example, the study procedure was explained to all eligible participants in lay terms and participants were allowed to ask questions for clarity. We reduced any socially desirable responses by not framing outcomes as good or bad, and the least socially desirable responses were often featured at the top of the list of responses. Participants were encouraged to attempt all questions truthfully, individually and to the best of their ability, and most recall questions involved short-term recall, usually a week or month.

### Ethical considerations

The research was conducted in accordance with the Declaration of Helsinki. We obtained ethical approval from the Faculty of Health Sciences Institutional Review Board of the University of Buea (IRB/FHS:901-01). In addition, we obtained administrative approvals from the Regional Delegation of Secondary Education, the Regional Delegation of Public Health for the Littoral Region, and the respective schools’ administrations to participate in the study. Written Informed Consent was obtained from all participants’ parents/guardians and assent was obtained from all participants after adequately informing them of any study inconvenience and their right to withdraw without prejudice at any stage during the study.

## Results

### Participants’ characteristics

A total of 645 adolescents submitted completed questionnaires. Participants’ mean age was 17 (± 1.52) years; 60.8% of them were girls, approximately 48% were in form five, and 71.7% (*n* = 428) were reading science subjects. Table 1 summarises the sociodemographic features of the study participants.

**Table 1:** Sociodemographic characteristics of senior state secondary school students in Douala, 2019

Variable	Frequency (%)
Number	645
Age (mean (SD))	17.01 (1.52)
Females (%)	392 (60.8)
Class (%)	
5th year	310 (48.1)
6th year	136 (21.1)
7th year	199 (30.9)
Science subjects (%)	428 (71.7)
Father’s education (%)	
None or Primary	62 (9.6)
Secondary	161 (25.0)
Tertiary	422 (65.4)
Mother’s education (%)	
None or Primary	81 (12.6)
Secondary	202 (31.4)
Tertiary	361 (56.1)
Guardian (%)	
Both Parents	422 (65.5)
One Parent	95 (14.8)
Other	127 (19.7)
Household size (median (IQR))	7 (5, 8)
Children in household (median (IQR))	4 (3, 6)

IQR = Interquartile range

### Knowledge and prevalence of NCD behavioural risk factors

Inadequate knowledge about NCDs and their risk factors was observed in almost half of the participants (Table 2). Three-quarters of all students reported alcohol consumption, with half starting as early as 13 years old. Among all participants, 42% were current alcohol consumers, and 4% consumed three or more alcoholic beverages daily. WHO recommendations for fruits and vegetable consumption and physical activity were not met. Over 93% of participants had unhealthy diets, while a fifth engaged in regular physical activity. Approximately 58.3% participated in weekly physical activity, and 49.8% spent at least 3 h daily on screens. Lifetime smoking prevalence was 11.3%, with a higher prevalence among males. Among those who experimented with smoking, 29% started by the age of 13. Overweight and obesity rates were 14.9% and 3.4%, respectively, with a higher prevalence among females. Elevated blood pressure was present in 4% of adolescents, more prevalent among boys.

**Table 2:** Prevalence of NCD behavioural risk factors in senior state secondary school students in Douala by gender

Variable		Overall	Female	Male	*p*-value
Number		645	392	253	
Inadequate knowledge (%)		314 (48.7)	196 (50.0)	118 (46.6)	0.452
Drinks alcohol (%)		270 (41.9)	155 (39.5)	115 (45.5)	0.16
Older than 13 at first alcoholic drink (%)		242 (37.5)	135 (34.4)	107 (42.3)	0.054
Three or more drinks/day when drinking (%)		24 (3.7)	13 (3.3)	11 (4.3)	0.644
Has been drunk or in trouble from drinking (%)		156 (24.2)	71 (18.1)	85 (33.6)	< 0.001
Poor diet (%)		599 (92.9)	361 (92.1)	238 (94.1)	0.425
Low physical activity (%)		510 (79.1)	319 (81.4)	191 (75.5)	0.09
Frequency of walk/cycle to school (%)					0.749
Everyday		364 (56.4)	219 (55.9)	145 (57.3)	
Never		149 (23.1)	89 (22.7)	60 (23.7)	
Somedays		132 (20.5)	84 (21.4)	48 (19.0)	
Frequency of school sports (%)					0.004
Most days		69 (10.7)	31 (7.9)	38 (15.0)	
Never		194 (30.1)	131 (33.4)	63 (24.9)	
Somedays		382 (59.2)	230 (58.7)	152 (60.1)	
Sedentary (%)		321 (50.0)	192 (49.2)	129 (51.2)	0.686
Has ever experimented with smoking (%)		73 (11.3)	30 (7.7)	43 (17.0)	< 0.001
Currently smokes (%)		19 (2.9)	8 (2.0)	11 (4.3)	0.146
Has high blood pressure (%)		26 (4.0)	9 (2.3)	17 (6.7)	0.01
Nutrition status (%)					< 0.001
Severely thin		5 (0.8)	2 (0.5)	3 (1.2)	
Thin		55 (8.5)	24 (6.1)	31 (12.3)	
Normal		467 (72.4)	264 (67.3)	203 (80.2)	
Overweight		96 (14.9)	80 (20.4)	16 (6.3)	
Obesity		22 (3.4)	22 (5.6)	0 (0.0)	

### Factors associated with behavioural risk factors

On multivariate regression analyses, alcohol drinking in adolescents was associated with maternal education (OR = 2.4; 95%CI = 1.3–4.7) (Table 3). Younger students were less likely to be sedentary (OR = 0.8; 95%CI = 0.7–0.9). Inadequate knowledge about NCDs and the male gender were associated with high blood pressure values (OR = 3.6; 95%CI = 1.4–10.3) and (OR = 3.6; 95%CI = 1.5–8.9), respectively. Male students were less likely to be overweight (OR = 0.2; 95%CI = 0.1–0.3).

**Table 3:** Correlations (aOR, 95% CI) of NCD behavioural risk factors in secondary school students in Douala, Cameroon

Determinants	Drinks alcohol	Good_diet	Physically inactive	Sedentary	Smokes	High blood	Overweight
Age	1.2 (1,1.5)	0.8 (0.6,1.1)	1.1 (0.9,1.3)	**0.8 (0.7,0.9)**	0.8 (0.4,1.8)	1.3 (0.9,1.9)	1 (0.8,1.2)
Boys (ref: girls)	0.9 (0.6,1.4)	2 (1,4.7)	0.7 (0.5,1.1)	1.2 (0.8,1.7)	0.9 (0.2,3.4)	**3.6 (1.5,8.9)**	**0.2 (0.1,0.3)**
Class (ref: 5th year)							
6th_year	0.9 (0.5,1.6)	1.5 (0.6,4)	1.1 (0.6,2)	1.7 (1,2.8)	1.9 (0.2,20.3)	0.8 (0.2,2.6)	0.5 (0.3,1)
7th_year	0.6 (0.3,1.1)	1.8 (0.7,4.8)	1.3 (0.7,2.4)	1.2 (0.7,1.9)	2.3 (0.3,18.9)	0.6 (0.2,1.8)	0.8 (0.4,1.5)
Science subject (ref: arts)	0.7 (0.5,1.2)	0.5 (0.2,1.2)	0.8 (0.4,1.2)	0.8 (0.5,1.2)	1.1 (0.2,6.5)	1.7 (0.6,5.7)	0.9 (0.5,1.5)
Father’s education (ref: < Primary)							
Secondary	1.6 (0.7,3.5)	.	1 (0.4,2.3)	1 (0.5,1.9)	.	1.9 (0.4,13.5)	1.5 (0.6,3.6)
Tertiary	1.8 (0.8,4)	.	0.9 (0.4,2)	1.4 (0.7,2.7)	.	1 (0.2,8.2)	1.1 (0.5,2.9)
Mother’s education (ref: < Primary)							
Secondary	**2.4 (1.3,4.7)**	0.2 (0,1.3)	0.8 (0.4,1.6)	1.4 (0.8,2.5)	.	1.3 (0.4,5.1)	0.5 (0.2,1)
Tertiary	1.9 (1,3.9)	0.4 (0,1.9)	0.9 (0.4,1.8)	1.2 (0.7,2.3)	.	0.7 (0.2,3.7)	0.8 (0.4,1.8)
Guardian (ref: both parents)							
One Parent	1.1 (0.6,1.9)	2.1 (0.7,9.1)	0.7 (0.4,1.2)	0.9 (0.6,1.5)	1.1 (0.2,5)	0.7 (0.1,2.8)	0.9 (0.5,1.6)
Other	0.7 (0.4,1.1)	1.9 (0.7,5.9)	0.7 (0.4,1.1)	0.6 (0.4,1)	.	1.8 (0.6,4.8)	0.9 (0.5,1.6)
Inadequate knowledge	1 (0.6,1.4)	1.1 (0.6,2.2)	1.3 (0.9,2)	1 (0.7,1.4)	1.1 (0.3,4.2)	**3.6 (1.4,10.3)**	0.7 (0.5,1.1)

## Discussion

### Main findings

This cross-sectional survey evaluated knowledge of NCDs, NCD behavioural risk factors, and sociodemographic determinants of risk factors among senior state secondary school adolescent students in an urban setting in Cameroon. The study contributes to understanding NCDs’ present and future burdens since there is a long lag between exposure to NCD risk factors and disease development, with most exposures occurring during adolescence.

The significance of this study is underpinned by the rising national trends in NCDs, which strain health systems already overwhelmed by infectious diseases. We found that about half of adolescents did not have adequate knowledge about NCDs and their risk factors. Only 20% of participants met recommended physical activity levels, nearly half lived sedentarily, and only 7% ate a healthy diet. Almost half of the participants drank alcohol during the month, while 3% reported cigarette smoking. Participants with inadequate knowledge of NCDs were more likely to have elevated blood pressure values, and being male increased the odds of having high blood pressure. Contrarily, being male appeared to be protective against overweight and obesity. The odds of being sedentary decreased with age. The odds of drinking alcohol seemed to grow with a higher maternal level of education.

### Study limitation

The study’s strengths include providing a broader assessment of NCD behavioural risk factors in adolescents using approaches that improve recall (requesting information from the recent past (7 days to a month), ensuring clarity and providing clarifications on questions asked). However, the study still has limitations. The reliance on self-filled questionnaires on behaviours happening over one month still subjects this study to substantial residual recall bias. We used non-validated criteria to assess some behavioural risk factors since strict WHO recommendations were difficult to employ in our setting. For example, we used a study-specific cutoff for healthy diet assessment. It was our understanding that participants eat on average three times a day: breakfast, lunch and supper. It is at these times that participants are expected to consume vegetables. As such, evaluating five servings of vegetables per the WHO recommendation proved challenging. Participants equally drew attention to the fact that they consume the same meal at lunch, supper, and sometimes for two days, thus making assessing vegetable consumption difficult. We, therefore, used the cutoff for adequate vegetable consumption as anyone who consumed vegetables daily. Study conclusions may not be generalisable to adolescents in the region as there may exist disparities between state and private schools and adolescents who do not attend school. Furthermore, we did not assess socioeconomic factors such as employment status and income, which have none negligible impact on health and behaviour because these metrics could not be answered accurately by the adolescents themselves. Assessing these factors would have required extending the questionnaire to the parents and guardians, and we were unsure about the response rate.

### Interpretation of findings

Approximately half of our study participants had inadequate knowledge of NCDs and their risk factors. Despite there being no significant difference between gender, other sociodemographic factors and adequate knowledge, we found that participants who had inadequate knowledge were three times more likely to have elevated blood pressure values for age and sex (OR = 3.6; 95%CI = 1.4–10.3). The prevalence of elevated blood pressure values was 4%, significantly more prevalent in boys. This distribution of NCD risk factors is similar to reports by Okpokowuruk et al. in Nigeria [18] and Noubiapa et al. in Cameroon [19]. They also reported increasing BMI as one factor significantly associated with elevated blood pressure in these populations. In our study, overweight and obesity were prevalent in 14.9% (*n* = 96) and 3.4% (*n* = 22) participants, and being male was found to be a protective factor (OR = 0.2; 95%CI = 0.1–0.3). Although many studies explain the associations between physical inactivity, unhealthy diet and overweight and obesity, these associations were not significant in our study. Unhealthy diets were highly prevalent and equally distributed in the study population with no associated factors.

Physical activity was low among participants, with just a fifth meeting the recommended levels of adequate physical activity. While participants conducted some activities, such as cycling every day (56%) and school physical education at least once a week (70%), almost half of them were still involved in substantial sedentary activities. The odds of being sedentary decreased with age (OR = 0.8; 95%CI = 0.7–0.9).

Alcohol consumption was prevalent among 55.3%, equally distributed by gender. However, a higher proportion of males consumed more than three alcoholic beverages daily, and males were likelier to get in trouble for drinking alcohol than females. Participants with higher maternal education levels were 2.4 times more likely to drink alcohol (95%CI = 1.3–47). This finding could be attributed to the fact that mothers with a higher level of education tend to have time-demanding jobs that limit child supervision. However, this is dissimilar to findings in Sri Lanka by Gamage et al. They reported a much lower prevalence of current alcohol consumption (11.5%), suggesting that parents with higher levels of education were more likely to advise their children on adopting healthy lifestyles and behaviours [20]. The higher prevalence of alcohol consumption could result from gaps in policy against alcoholism among young people and specifically around the school milieu. In addition, the extensive publicity and marketing of the alcohol industries create an environment conducive to excessive and uncontrolled consumption of alcohol [21]. Finding ways to effectively prohibit the purchase of alcoholic beverages by children and adolescents remains a nationwide challenge.

We found that 11.3% of participants had experimented with cigarette smoking; 25.3% were current smokers. This proportion was evenly distributed by gender. Forty-two per cent of participants were exposed to secondhand smoke. Although we found the prevalence of cigarette smoking to be relatively low (2.9%), we postulate that the problem of cigarette smoking is similar to that of alcohol, especially with prohibiting purchases by children and adolescents, despite how dissuasive the label warnings have been thought to be [21]. There were no factors found to be significantly associated with cigarette smoking. A study by Ngahane et al. in Douala, Cameroon [22] found a higher prevalence of cigarette smoking amongst adolescents and young adults. They found the male gender, increasing age and parental smoking to be significant correlates. Our findings could differ from these because of the different sampling methods employed.

### Implications

Our study highlights that the risk factors for NCDs already abound in adolescents. Though NCDs take a long time to develop, the high prevalence of risk factors indicates that our participants are well on their way to escalating the future NCD burden. Since these are primarily behavioural risk factors, there is an opportunity to design public health measures such as incorporating healthy living in school curriculums while improving environments that influence adolescent behaviour. The emphasis on the environment is paramount since adolescents have a lesser choice over their habitats, and the decision is in the hands of the parents/guardians. There is a need for more research to establish the transition of adolescent risk factors to adult disease through life course approaches in these settings.

## Conclusion

Our cross-sectional survey shows inadequate knowledge about NCDs and their risk factors and a high prevalence of NCD behavioural risk factors among adolescents attending state secondary schools in an urban setting in Cameroon. This pattern predicts a higher burden of future adult NCDs in the country, flagging the need for urgent public health interventions for this age group. Further research is needed to establish the transition of adolescent risk factors to adult disease through life course approaches in these settings.

## Data Availability

Data are available upon request from the corresponing author.
